# Enhanced antibacterial activity of TiO_2_ nanoparticle surface modified with *Garcinia zeylanica* extract

**DOI:** 10.1186/s13065-017-0236-x

**Published:** 2017-01-12

**Authors:** U. L. N. H. Senarathna, S. S. N. Fernando, T. D. C. P. Gunasekara, M. M. Weerasekera, H. G. S. P. Hewageegana, N. D. H. Arachchi, H. D. Siriwardena, P. M. Jayaweera

**Affiliations:** 1 Department of Microbiology, Faculty of Medical Sciences, University of Sri Jayewardenepura, Colombo, Sri Lanka; 2 Department of Nidana Chikitsa, Institute of Indigenous Medicine, University of Colombo, Colombo, Sri Lanka; 3 Department of Chemistry, University of Sri Jayewardenepura, Colombo, Sri Lanka; 4 Department of Optoelectronics and Nanostructure Science, Graduate School of Science and Technology, Shizuoka University, Hamamatsu, Japan

**Keywords:** Titanium dioxide, Antibacterial, Methicillin-resistant *Staphylococcus aureus*, *Garcinia*

## Abstract

**Background:**

The antibacterial activity of 21 nm TiO_2_ nanoparticles (NPs) and particles modified with *Garcinia zeylanica* (*G. zeylanica*) against Methicillin resistant *Staphylococcus aureus* was investigated in the presence and absence of light.

**Results:**

Surface modification of TiO_2_ NPs with the adsorption of *G. zeylanica* extract, causes to shift the absorption edge of TiO_2_ NPs to higher wavelength. TiO_2_ NPs, *G. zeylanica* pericarp extract showed significant bactericidal activity which was further enhanced in contact with the TiO_2_ modified *G. zeylanica* extract.

**Conclusions:**

The antimicrobial activity was enhanced in the presence of TiO_2_ NPs modified with *G. zeylanica* and with longer contact time.

## Background

Nanotechnology is a nascent technology, gaining popularity globally due to its usefulness in various fields. Nanometals ranging from 1 to 100 nm in size have unique physical and chemical properties which can be exploited for various applications [1, 2]. Further these are promising novel therapeutic agents having antimicrobial and antibiofilm activity.

Development of microbial resistance to antibiotics is a major challenge in the medical field. Therefore, the search for drugs with new modes of action is of major interest in the pharmaceutical and research communities. Two potential sources of novel antimicrobial agents are medicinal plants and nanomaterials [3, 4]. The antimicrobial properties of nanomaterials including metal nanoparticles can be attributed to different mechanisms such as generation of reactive oxygen species, inactivation of cellular enzymes and nucleic acids of the microbes resulting in pore formation in the bacterial cell wall [3]. Among the metal nanoparticles TiO_2_ NPs are known to be cost effective, stable and safe for humans and the environment. A unique property of TiO_2_ NPs is the photocatalytic property resulting in enhanced microbicidal activity on exposure to light in the UV range [3, 5]. TiO_2_ NPs exist in three crystalline phases, where the anastase phase demonstrates high photocatalytic and antimicrobial properties [3].

*Garcinia zeylanica* is an endemic plant to Sri Lanka, which belongs to the family Guttiferae (Clusiaceae). Ragunathan et al. [6] reported antibacterial activity of pericarp of *G. zeylanica* extract against MRSA, while it had no antimicrobial activity against *Candida albicans* and *Candida parapsilosis* [7]. Others have reported antimicrobial activity of Garcinia species against *Staphylococcus aureus*, *Streptococcus pyogenes* and some Gram negative bacteria [8]. *Garcinia* species have many important phytochemicals with antimicrobial potential [9, 10]. The phytochemical analysis of *G. zeylanica* which is an endemic plant to Sri Lanka, is not yet documented. This study aimed to determine the antibacterial activity of TiO_2_ NPs modified with *G. zeylanica* aqueous extract. The combined synergistic effect of phytochemicals and TiO_2_ NPs were also investigated.

## Methods

### Preparation of *Garcinia zeylanica* aqueous extract

Dried pericarp of *G. zeylanica* was collected locally and authenticated at the Bandaranayaka Memorial Ayurveda Research Institute, Navinna, Maharagama, Sri Lanka. The pericarp was rinsed, dried (6 h at 42 °C) and aqueous extract was prepared using 30 g of plant material in 720 ml distilled water, then boiled under low heat to reduce the volume to 120 ml according to Ayurvedic protocol [11]. The plant extract was filtered using sterile Whatman No 1 filter paper. The filtrate was transferred to a sterile glass container and stored in the refrigerator (4 °C) up to 2 weeks.

### Characterization and surface modification of TiO_2_ NPs with *G. zeylanica* extract

Surface modification of 21 nm TiO_2_ NPs (Sigma Aldrich) with *G. zeylanica* aqueous extract was done by refluxing 25 ml of *G. zeylanica* aqueous extract with 0.30 g of TiO_2_ (mainly anatase). Solid part was centrifuged and separated. Separated solid was washed with distilled water several times by centrifugation. Washed solid was separated air dried and placed in a vacuum desiccator for 48 h.

Scanning electron microscope (SEM) imaging was performed to understand the surface morphology of TiO_2_ of the coated petri dishes. SEM imaging was done using FE-SEM (JSM-6320F) at accelerating voltages of 10 kV. Powered X-ray diffraction (XRD) analysis was carried out for the identification of the phase of coated TiO_2_ using Ultima III (Rigaku) powder diffractometer (Cu-Kα/λ = 0.154 nm). Surface characterization of pure and modified NPs were performed using diffuse reflectance spectroscopy and attenuated total reflectance-Fourier transform infrared spectroscopy (ATR-FTIR). Diffuse reflectance spectroscopic studies were carried out using PerkinElmer Lambda 35 spectrophotometer equipped with integrating sphere. ATR-FTIR analysis was carried out using Thermo Scientific Nicolet iS10 FTIR spectrometer.

### Phytochemical analysis of the aqueous *G. zeylanica* extract

Qualitative analysis of various phytocompounds present in the *G. zeylanica* aqueous extract was done using previously described protocol by Krishnamoorty et al. [12]. Flavanoids, terpenoids, phenols, tannins, cardiac glycosides, carbohydrates, saponins, amino acids, phlobatannin, sterols and alkaloids were detected in this study.

### Microorganisms

A clinically confirmed isolate of Methicillin resistant *S. aureus* was obtained from the culture collection at the Department of Microbiology, University of Sri Jayewardenepura. The organism was cultured on Nutrient agar at 37 °C for 18 h. Suspensions of organisms were prepared in sterile normal saline to obtain a 0.5 MacFarland absorbance corresponding to 10^8^ organisms/ml.

### Determination of antimicrobial activity of 21 nm TiO_2_ NPs, and TiO_2_ NPs modified with *G. zeylanica*

TiO_2_ NPs was used at a concentration of 13.9 g/l in sterile miliq (MQ) water [13]. Suspension of TiO_2_ was prepared by sonication at 35 kHz for 1 h followed by autoclaving for 30 min at 121 °C. The pH of all solutions was adjusted to pH 5.5 prior to coating of the petri dishes.

A separate plate (A) was used as negative control which contained MQ water. Sterile 3 cm petri dishes were coated with (B) TiO_2_ only, (C) *G. zeylanica* aqueous extract only and (D) *G. zeylanica* extract modifies with TiO_2_. Each petri dish was coated by adding 1 ml of solutions of B, C and D to individual petri dishes. The petri dishes were then evaporated to dryness.

One milliliter of MRSA suspension (10^8^ organisms/ml) was added to each petri dish. The inoculated petri dishes were kept for 1, 4 and 24 h, at room temperature. At the end of each time point 100 μl of suspension was collected from each petri dish and colony forming units/ml (CFU/ml) was determined by spread plate method on Nutrient agar. Further, to determine the enhanced antimicrobial activity due to the photocatalytic activity of TiO_2_ NPs, one set of petri dishes (tests and control) were incubated for 30 min in sunlight after addition of MRSA suspension and the number of colonies were counted as described above. All experiments were done in triplicates.

### Statistical analysis

Colony forming units/ml was calculated by multiplying the number of colonies obtained by plating 100 μl of suspension by the dilution factor. This was further multiplied by 10 to obtain CFU/ml. The percentage reduction was calculated as follows:

$${\text{Average}}\;{\text{reduction}}\% = \frac{{{\text{CFU/ml}}\;{\text{in}}\;{\text{MQ}} - {\text{CFU/ml}}\;{\text{in}}\;{\text{TiO}}_{2} }}{{{\text{CFU/ml}}\;{\text{in}}\;{\text{MQ}}}} \times 100$$

The paired *t* test was used to compare the significant differences between test and control. Significance was tested at p = 0.05.

## Results and discussion

### SEM and XRD analysis

**Fig. 1:**
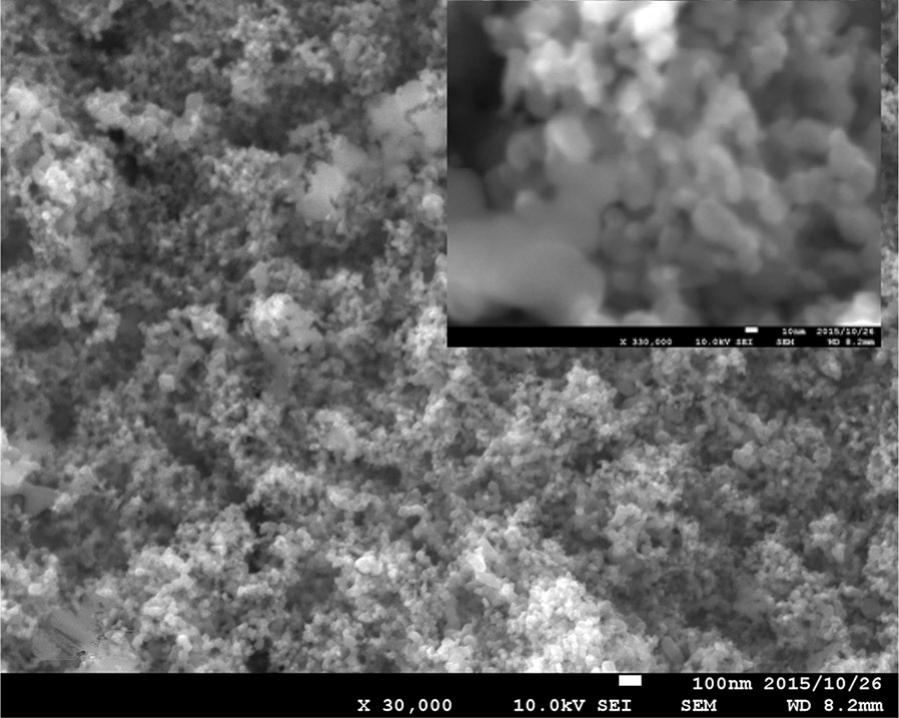
SEM image of TiO_2_ coated on a petri dish. *Inset* 10 nm magnification

**Fig. 2:**
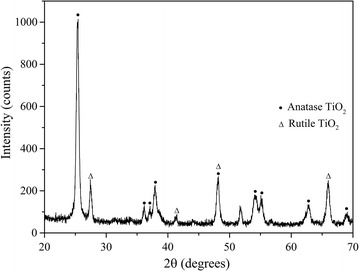
XRD pattern of TiO_2_ NPs

A scanning electron microscope (SEM) image of the surface of TiO_2_ coated petri dish is shown in the Fig. 1. Petri dish surface was evenly coated with TiO_2_. Figure 2 shows the XRD pattern of the coated TiO_2_. The pattern recorded closely resembles the previously published XRD pattern of the anatase phase and rutile phase of TiO_2_ [14–16].

### Diffuse reflectance, UV–visible and ATR-FTIR study

**Fig. 3:**
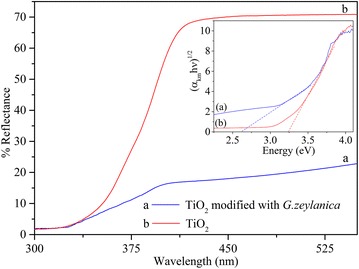
Diffuse reflectance spectra of *a* TiO_2_ modified with *G. zeylanica* extract and *b* TiO_2_. *Inset* Kubelka–Munk transformed reflectance spectra

**Fig. 4:**
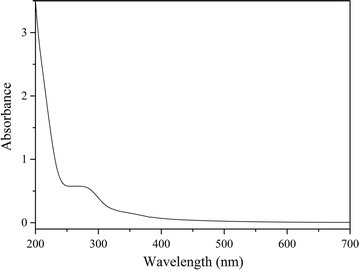
UV–Vis absorption spectrum of aqueous extract of *G. zeylanica*

Diffuse reflectance spectra of TiO_2_ and TiO_2_ modified with *G. zeylanica* aqueous extract are shown in Fig. 3. Alteration of the diffuse reflectance spectrum of TiO_2_ noticeably indicates the characteristic change of TiO_2_ surface followed by the adsorption of *G. zeylanica* extract. The diffuse reflectance spectra were analyzed using [17] the Kubelka–Munk transformed reflectance spectra according to,

$$\alpha_{KM} = \frac{{\left( {1 - R_{\infty } } \right)^{2} }}{{2R_{\infty } }}$$

where *α*_*KM*_ is the equivalent absorption coefficient, *R*_*∞*_ is the reflectance of an infinitely thick sample with respect to a reference at each wavelength. Kubelka–Munk transformed reflectance spectra are shown in the inserted image of Fig. 3. Surface modification of TiO_2_ NPs with the adsorption of *G. zeylanica* extract, causes to decrease the band gap energy of TiO_2_ NPs. Band gap energy of bare TiO_2_ and *G. zeylanica* extract adsorbed TiO_2_ were found to be 3.24 and 2.61 eV, respectively. Lowering the band gap energy of TiO_2_ is leading to enhancement of photocatalytic activity under visible light [18] which is reflected by change in the colour of the TiO_2_ surface to buff colour. UV–visible absorption spectrum of dilute solution of *G. zeylanica* aqueous extract is shown in the image of Fig. 4.

**Fig. 5:**
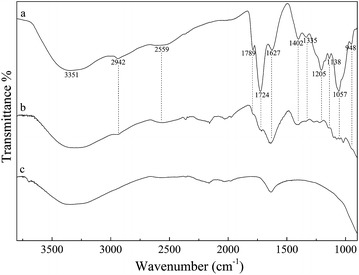
ATR-FTIR spectra of *a* dried *G. zeylanica* extract, *b* TiO_2_ modified with *G. zeylanica* extract, and *c* TiO_2_

ATR-FTIR spectra of dried pulp of *G. zeylanica* extract, *G. zeylanica* extract adsorbed TiO_2_ and TiO_2_ are shown in Fig. 5. ATR-FTIR spectrum of dried pulp of *G. zeylanica* extract closely resembles the previously published FTIR spectrum of dried pulp of *G. pedunculata* [19]. Adsorption of surface anchoring compounds in *G. zeylanica* extract on to TiO_2_ is confirmed by the presence of IR peaks of *G. zeylanica* extract, for *G. zeylanica* extract treated TiO_2_. FTIR frequencies suggested that the presence of –OH group (3351 cm^−1^ for O–H stretching), alkane side chains (2942 cm^−1^ is characteristic for C–H stretching), carbonyl group (1724 cm^−1^ for the C=O stretching), and carboxylic group (1402 cm^−1^ is for (COO^−^) asymmetric stretching) [19–21]. IR absorption peak at 1724 cm^−1^ is decreased by the adsorption of *G. zeylanica* extract into TiO_2_, which may be due to the deprotonating of carboxylic group [20].

### Phytochemical screening of the aqueous extract of *G. zeylanica*

Qualitative analysis of *G. zeylanica* extract revealed the presence of tannins, cardiac glycosides, carbohydrates, coumarin and saponins (Table 1). Tanins are a group of polyphenolic compounds and their antimicrobial activity against fungi, bacteria and viruses have been reported [22]. Coumarins which are reported to be present in plant extracts including *Garcinia* species, have antimicrobial and anti-inflammatory activities [23]. Saponin is a glycoside and are present in plants with reported antibacterial and antifungal activity [24].

**Table 1:** Phytochemical screening of the aqueous extract of *G. zeylanica*

Phytoconstituents	Test/reagents	Observation
Alkaloids	Mayer’s test	Negative
Tannins	Braymer’s test	Positive
Saponins	Foam test	Positive
Anthraquinones	Benzene, 10% NH_3_	Negative
Flavanoids	1% aluminium solution	Negative
Carbohydrates	Molisch’s test	Positive
Amino acids	Ninhydrin test	Negative
Steroids	Salkowski test	Negative
Terpenoids	Salkowski test	Negative
Cardiac glycosides	FeCl_3_, conc. H_2_SO_4_	Positive
Coumarin	Alcoholic NaOH	Positive

### Antibacterial activity of TiO_2_

**Fig. 6:**
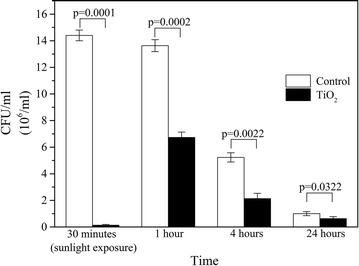
Antibacterial activity of TiO_2_ against MRSA

The colony forming units of MRSA reduced significantly (p = 0.0001) after 30 min in the presence of TiO_2_ following sunlight exposure compared to the control having only MQ water exposed to sunlight. When MRSA suspension (10^8^ organisms/ml) was added to TiO_2_ coated plates and incubated for 1, 4 and 24 h (without exposure to sunlight), there was a significant reduction in the colony counts (p = 0.0002, 0.0022, 0.0322 respectively) when compared to the control (Fig. 6). The average percentage reduction of MRSA was seen to be 99.1% after 30 min sunlight exposure when compared to the control. The percentage reduction of colony counts seen after 1, 4 and 24 h, were 48.3, 59.2 and 32.9% respectively. These results demonstrate that TiO_2_ itself has antimicrobial activity which is enhanced in the presence of sunlight. TiO_2_ has photocatalytic properties which have been reported to be useful as a microbicide [3]. Our study shows that in the presence of sunlight the antimicrobial activity of TiO_2_ is enhanced against MRSA. Several groups have evaluated the antimicrobial activity of TiO_2_ against both Gram negative bacteria such as *Escherichia coli* [3], *Salmonella typhimurium* [4], *Pseudomonas aeruginosa* [4, 25], *Bacteroides fragilis* [4] and Gram positive bacteria such as *S. aureus* [25], *Enterococcus faecalis* [26], *Streptococcus pneumoniae* [26], MRSA [26], fungi such as *C. albicans* [27], *Aspergillus niger* and *Trichoderma reesei* [28] and viruses such as HSV-1 [29] and influenza virus [30]. The advantage of TiO_2_ as an environmental disinfectant is mainly due to its photocatalytic activity in the presence of UV irradiation. TiO_2_, when exposed to light in the UV range (λ < 400 nm) result in generation of redox reactions that produce reactive oxygen species, such as hydroxyl radical (·OH), superoxide radical (·O_2_^−^) and singlet oxygen (^1^O_2_). These free radicals contribute to the biocidal activity by destruction of cellular organic compounds [26]. Hence close proximity of the microorganisms to the TiO_2_ NPs is needed for good bactericidal activity.

The antimicrobial activity of TiO_2_ even in the absence of photo activation has been well reported [26]. TiO_2_ carries a positive charge while the surface of microorganisms carry negative charges resulting in an electromagnetic attraction between microorganisms and the TiO_2_ NPs which leads to oxidation reactions. TiO_2_ deactivates the cellular enzymes and DNA by coordinating to electron-donating groups, such as: thiols, amides, carbohydrates, indoles, hydroxyls etc. The resulting pits formed in bacterial cell walls lead to increased permeability and cell death [26].

TiO_2_ NPs are reported to be non carcinogenic and nontoxic [31] and are used extensively in food packaging [5], textile industry [32], self-cleaning ceramics and glass [33], in the paper industry for improving the opacity of paper [33], cosmetic products such as sunscreen creams [33] etc. Further, TiO_2_ NPs are used in commercial products such as water purification plants [34]. The antimicrobial activity of TiO_2_ NPs are exploited in medical devices, in order to prevent biofilm formation and sepsis [35–37].

### Antibacterial effect of *G. zeylanica* aqueous extract

**Fig. 7:**
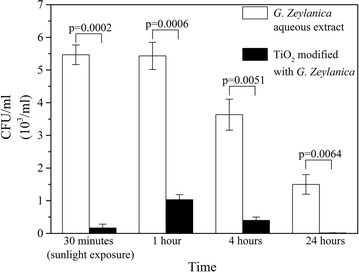
Antibacterial activity of *G. zeylanica* aqueous extract and TiO_2_ modified with *G. zeylanica* aqueous extract

Antimicrobial activity of *G. zeylanica* alone and TiO_2_ modified with *G. zeylanica* showed a significant reduction in colony forming units at all time points tested as shown in Fig. 7. When MRSA was treated with the aqueous extract of *G. zeylanica* (0.25 g/ml) and exposed to sunlight for 30 min, a significant reduction of MRSA colony counts were observed, compared to the control (p = 0.0001). Further, when MRSA was incubated without sunlight for 1, 4 and 24 h, a significant reduction (p = 0.0002, 0.0007, 0.0044 respectively) of colony counts was seen compared to the control. This shows that the plant extract itself exhibits strong antimicrobial activity against MRSA. The average percentage reduction of MRSA was seen to be 99.96% after 30 min sunlight exposure when compared to the control. The percentage reduction of colony counts seen after 1, 4 and 24 h, without sunlight were 99.96, 99.93 and 99.84% respectively. The TiO_2_ modified with *G. zeylanica* aqueous extract demonstrated remarkably enhanced antimicrobial activity compared to the antimicrobial activity of TiO_2_ alone. Dried pericarp of *G. zeylanica* and other *Garcinia* species is widely used as a flavouring and preserving agent in traditional culinary practices in Sri Lanka and other Asian countries. In Ayurvedic practices, *Garcinia* is used in treatment of skin and soft tissue infections. Further, it is included as a component of Ayurvedic wound wash. In this study, the aqueous extract of the pericarp of an endemic plant, *G. zeylanica* was investigated for synergistic microbicidal activity when combined with TiO_2_ NPs. While the antimicrobial activity of other *Garcinia* species have been reported in detail, reports on the antimicrobial activity of *G. zeylanica* is not available. Recent study by Ragunathan reports that the aqueous extract of *G. zeylanica* pericarp showed antibacterial activity against MRSA while no activity was detected for Candida species [6]. The *G. zeylanica* aqueous extract was used after adjusting the pH to 5.5 throughout the experiments, which is compatible for use as a wound wash.

*Garcinia zeylanica* extracts from other species have been reported to contain hydroxy citric acid, xanthones, flavonoids and benzophenone derivatives such as garcinol [38]. Previous reports have investigated the antimicrobial activity of *Garcinia Cambogia* [39], and *Garcinia indica* [40].

### Antibacterial effect of TiO_2_ modified with *G. zeylanica* aqueous extract

When the TiO_2_ was modified with *G. zeylanica* extract, there was significant antimicrobial activity in the presence of sunlight (p value = 0.0001) compared to the control. When the modified extract was incubated with MRSA for 1, 4 and 24 h, the antimicrobial activity was seen to be further enhanced with increasing incubation time (p = 0.0002, 0.0007, 0.0044). The percentage reduction of colony counts at all four time points were >99.99%. These results show that the antimicrobial activity of TiO_2_ was significantly enhanced when modified with *G. zeylanica* both in the presence and absence of sunlight as shown in Fig. 7. Exposure to sunlight and prolong contact was seen to further enhance the antimicrobial activity.

**Fig. 8:**
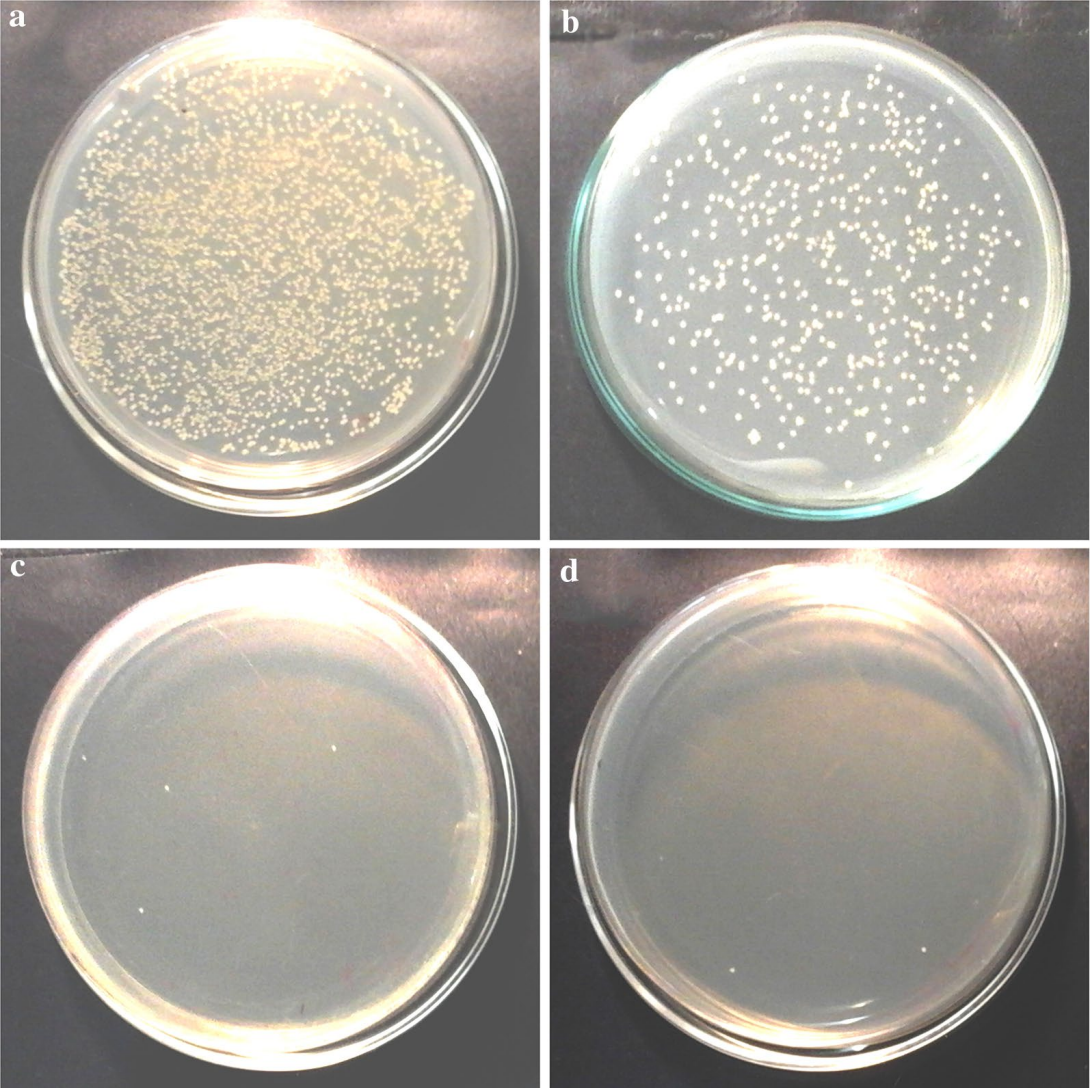
MRSA colonies with 1 h incubation **a** MQ water, **b** TiO_2_, **c** *G. zeylanica* aqueous extract, and **d** TiO_2_ modified with *G. zeylanica* aqueous extract

On comparison of antimicrobial activity of *G. zeylanica* extract only and TiO_2_ modified with *G. zeylanica* aqueous extract, a significant enhancement of microbicidal activity was observed in the presence of TiO_2_ modified with *G. zeylanica* aqueous extract (exposed to sunlight or without sunlight exposure). Further, prolonged contact with TiO_2_ modified with *G. zeylanica* aqueous extract showed a significant reduction in colony counts compared to *G. zeylanica* alone as shown in Table 2. Figure 8 shows a representative experiment where colony counts were obtained after 1 h contact of MRSA (10^8^ cells/ml) with the control (a), TiO_2_ coated plate (b), *G. zeylanica* aqueous extract coated plate (c) and TiO_2_ modified with *G. zeylanica* aqueous extract coated plate (d). A clear reduction in colony counts were observed in plates c (99.96%) and d (99.99%) when compared to the control. The antimicrobial activity of TiO_2_ modified with *G. zeylanica* aqueous extract is thought to be due to multiple mechanisms of the phytochemicals and TiO_2_ NPs. Garcinol which is an important phytochemical, is reported to competitively inhibit histone acetyltransferases in cells [10]. It has also been reported to regulate gene expression in HeLa cells. Further, garcinol is able to induce apoptosis in cells making it a potential therapeutic agent in cancer treatment [10]. The combination of *G. zeylanica* and TiO_2_ as a potential antimicrobial agent in medicine may be an important future direction due to the widely reported emergence of multidrug resistance among microbes, which is a major challenge in medicine.

**Table 2:** Comparison of antimicrobial activity of *G. zeylanica* extract and TiO_2_ modified with *G. zeylanica* aqueous extract

Time	*G. zeylanica* aqueous extract (CFU/ml)	TiO_2_ modified with *G. zeylanica* aqueous extract (CFU/ml)	p value
After 30 min sunlight exposure	5467	167	0.0002
After 1 h incubation period	5433	1033	0.0006
After 4 h incubation period	3633	400	0.0051
After 24 h incubation period	1500	13	0.0064

## Conclusions

 Anatase 21 nm TiO_2_ NPs shows antimicrobial activity against MRSA following photoactivation by sunlight. *G. zeylanica* aqueous extract itself has antimicrobial activity against MRSA. Enhanced antimicrobial activity was observed when the TiO_2_ was modified with *G. zeylanica* aqueous extract. Activity against MRSA was further enhanced when TiO_2_ was modified with *G. zeylanica* aqueous extract with the exposure to the sunlight.

## References

[1] Horikoshi S, Serpone N (2013). Introduction to nanoparticles. Microwaves in nanoparticle synthesis.

[2] Hasan S (2015). A review on nanoparticles: their synthesis and types. Res J Recent Sci.

[3] Ahmad R, Sardar M (2013). TiO_2_ nanoparticles as an antibacterial agents against *E. coli*. Int J Innov Res Sci Eng Technol.

[4] Hajipour MJ, Fromm KM, Ashkarran AA, Jimenez de Aberasturi D, Larramendi IRd, Rojo T (2012). Antibacterial properties of nanoparticles. Trends Biotechnol.

[5] Othman SH, Abd Salam NR, Zainal N, Kadir Basha R, Talib RA (2014). Antimicrobial activity of TiO_2_ nanoparticle-coated film for potential food packaging applications. Int J Photoenergy.

[6] Ragunathan K, Radhika N, Gunathilaka D, Weerasekera M, Hewageegana S, Fernando S, et al (2015) Antimicrobial activities of selected herbs and two herbal decoctions against methicillin resistant *Staphylococcus aureus* (MRSA). In: Proceedings of annual scientific sessions of faculty of medical sciences, p 36

[7] Radhika ND, Gunathilaka DP, Ragunathan K, Gunasekara TD, Weerasekara MM, Fernando SS, Arawwawala LAD, Hewageegana S (2015) Antifungal activities of selected plant extracts against *Candida albicans* and *Candida parapsilosis*. In: Engineering social transformation through research and development proceedings of annual research symposium, pp 68–69

[8] Seanego CT, Ndip RN (2012). Identification and antibacterial evaluation of bioactive compounds from *Garcinia kola* (Heckel) seeds. Molecules.

[9] Tharachand SI, Avadhani M (2013). Medicinal properties of malabar tamarind [*Garcinia cambogia* (Gaertn) DESR]. Int J Pharm Sci Rev Res.

[10] Hemshekhar M, Sunitha K, Santhosh MS, Devaraja S, Kemparaju K, Vishwanath B (2011). An overview on genus *Garcinia*: phytochemical and therapeutical aspects. Phytochem Rev.

[11] Pandit Shastri P (1920) Uttara khanda. In: Sharangadhara Samhita. Pandurang Jawaji, Bombay, pp 353–354

[12] Krishnamoorthy V, Nagappan P, Sereen AK, Rajendran R (2014). Preliminary phytochemical screening of fruit rind of *Garcinia cambogia* and leaves of *Bauhinia variegate*—a comparative study. Int J Curr Microbiol Appl Sci.

[13] Verdier T, Coutand M, Bertron A, Roques C (2014). Antibacterial activity of TiO_2_ photocatalyst alone or in coatings on *E. coli*: the influence of methodological aspects. Coatings.

[14] Kim TK, Lee MN, Lee SH, Park YC, Jung CK, Boo JH (2005). Development of surface coating technology of TiO_2_ powder and improvement of photocatalytic activity by surface modification. Thin Solid Films.

[15] Chang M, Song Y, Zhang H, Sheng Y, Zheng K, Zhou X (2015). Hydrothermal assisted sol-gel synthesis and multisite luminescent properties of anatase TiO_2_:Eu^3+^ nanorods. RSC Adv.

[16] Lee CH, Rhee SW, Choi HW (2012). Preparation of TiO_2_ nanotube/nanoparticle composite particles and their applications in dye-sensitized solar cells. Nanoscale Res Lett.

[17] Reyes-Coronado D, Rodriguez-Gattorno G, Espinosa-Pesqueira ME, Cab C, de Coss R, Oskam G (2008). Phase-pure TiO_2_ nanoparticles: anatase, brookite and rutile. Nanotechnology.

[18] Luo X, Deng F, Min L, Luo S, Guo B, Zeng G (2013). Facile one-step synthesis of inorganic-framework molecularly imprinted TiO_2_/WO_3_ nanocomposite and its molecular recognitive photocatalytic degradation of target contaminant. Environ Sci Technol.

[19] Mudoi T, Deka D, Devi R (2012). In vitro antioxidant activity of *Garcinia pedunculata*, an indigenous fruit of North Eastern (NE) region of India. Int J PharmTech Res.

[20] Mudunkotuwa IA, Grassian VH (2010). Citric acid adsorption on TiO_2_ nanoparticles in aqueous suspensions at acidic and circumneutral pH: surface coverage, surface speciation, and its impact on nanoparticle–nanoparticle interactions. J Am Chem Soc.

[21] See I, Ee GC, Teh SS, Kadir AA, Daud S (2014). Two new chemical constituents from the stem bark of *Garcinia mangostana*. Molecules.

[22] Scalbert A (1991). Antimicrobial properties of tannins. Phytochemistry.

[23] Cowan MM (1999). Plant products as antimicrobial agents. Clin Microbiol Rev.

[24] Pistelli L, Bertoli A, Lepori E, Morelli I, Panizzi L (2002). Antimicrobial and antifungal activity of crude extracts and isolated saponins from *Astragalus verrucosus*. Fitoterapia.

[25] Gupta K, Singh RP, Pandey A, Pandey A (2013). Photocatalytic antibacterial performance of TiO_2_ and Ag-doped TiO_2_ against *S. aureus*, *P. aeruginosa* and *E. coli*. Beilstein J Nanotechnol.

[26] Nakano R, Hara M, Ishiguro H, Yao Y, Ochiai T, Nakata K (2013). Broad spectrum microbicidal activity of photocatalysis by TiO_2_. Catalysts.

[27] Yang JY (2006). Photocatalytic antifungal activity against *Candida albicans* by TiO_2_ coated acrylic resin denture base. J Korean Acad Prosthodont.

[28] Durairaj B, Muthu S, Xavier T (2015). Antimicrobial activity of *Aspergillus niger* synthesized titanium dioxide nanoparticles. Adv Appl Sci Res.

[29] Markov SL, Vidaković AM (2014). Testing methods for antimicrobial activity of TiO_2_ photocatalyst. Acta Period Technol.

[30] Nakano R, Ishiguro H, Yao Y, Kajioka J, Fujishima A, Sunada K (2012). Photocatalytic inactivation of influenza virus by titanium dioxide thin film. Photochem Photobiol Sci.

[31] Runa S, Khanal D, Kemp ML, Payne CK (2016). TiO_2_ nanoparticles alter the expression of peroxiredoxin anti-oxidant genes. J Phys Chem C.

[32] Senic Z, Bauk S, Vitorovic-Todorovic M, Pajic N, Samolov A, Rajic D (2011). Application of TiO_2_ nanoparticles for obtaining self-decontaminating smart textiles. Sci Tech Rev.

[33] AZoNano (2013) Titanium oxide (Titania, TiO_2_) nanoparticles—properties, applications. Retrieved from: http://www.azonano.com/article.aspx. ArticleID=3357

[34] Cermenati L, Pichat P, Guillard C, Albini A (1997). Probing the TiO_2_ photocatalytic mechanisms in water purification by use of quinoline, photo-fenton generated OH radicals and superoxide dismutase. J Phys Chem B.

[35] Gupta SM, Tripathi M (2011). A review of TiO_2_ nanoparticles. Chin Sci Bull.

[36] Ravishankar Rai V, Jamuna Bai A, Mendez-Vilas A (2011). Nanoparticles and their potential application as antimicrobials. Science against microbial pathogens: communicating current research and technological advances.

[37] Arora H, Doty C, Yuan Y, Boyle J, Petras K, Rabatic B (2010). Titanium dioxide nanocomposites. Nanomaterials for the life sciences (series nr. 8).

[38] Tharachand C, Selvaraj CI, Abraham Z (2015). Comparative evaluation of anthelmintic and antibacterial activities in leaves and fruits of *Garcinia cambogia* (Gaertn.) desr. and *Garcinia indica* (Dupetit-Thouars) choisy. Braz Arch Biol Technol.

[39] Jayarathne TU, Vidanarachchi JK, Kalubowila A, Himali SMC (2014) Antioxidant and antimicrobial effect of *Garcinia cambogia* and *Tamarindus indica* on minced nematalosa galatheae fish under refrigerated storage. In: Proceedings of the Peradeniya University International Research Sessions (iPURSE 2014), vol 18, Sri Lanka, p 211

[40] Sutar R, Mane S, Ghosh J (2012). Antimicrobial activity of extracts of dried kokum (*Garcinia indica* C). Int Food Res J.

